# Clinical, laboratory, and therapeutic characteristics of cutaneous leishmaniasis: 15-year incidence in Crete and Greece, and implications for clinician awareness

**DOI:** 10.3205/dgkh000636

**Published:** 2026-03-02

**Authors:** Aristos Aristodimou, Evangelos I. Kritsotakis, Maria Antoniou, Konstantinos Krasagakis, Georgios Evangelou, Achilleas Gikas

**Affiliations:** 1 Division of Internal Medicine, School of Medicine, University of Crete, Heraklion, Crete, Greece; 2 Internal Medicine Department, Limassol General Hospital, State Health Organization Services, Limassol, Cyprus; 3 Laboratory of Biostatistics, School of Medicine, University of Crete, Heraklion, Crete, Greece; 4 Parasitology and Medical Entomology Unit, School of Medicine, University of Crete, Heraklion, Crete, Greece; 5 Dermatology Department, University Hospital of Heraklion, Heraklion, Greece

**Keywords:** cutaneous leishmaniasis, visceral leishmaniasis, Crete, Leishmania tropica, Leishmania infantum, clinician awareness

## Abstract

**Background:**

Cutaneous leishmaniasis (CL) is an under-recognised parasitic disease in southern Europe, where heterogeneous clinical presentations and limited clinician familiarity can contribute to delayed diagnosis.

**Method:**

We conducted a 15-year retrospective case-series study at a tertiary-care referral hospital in Crete, Greece, to describe the epidemiological, clinical, laboratory and therapeutic characteristics of confirmed CL cases.

**Results:**

Twenty-five patients were identified, including two with concomitant visceral leishmaniasis. Most presented with solitary, painless lesions on exposed sites, and the median duration of skin lesions before diagnosis was 327 days, highlighting a substantial diagnostic delay. PCR was the most sensitive diagnostic tool, followed by histopathology and culture, while species identification confirmed *Leishmania tropica* in selected CL cases.

Treatment approaches varied, including systemic agents, topical therapies and observation. Failure and relapse rates were 12% and 16%, respectively, and occurred mainly in patients with significant comorbidities or immunosuppression.

**Conclusion:**

The island of Crete is a persistent epicenter of CL in Greece, with a low apparent incidence but notable clinical complexity. Delayed recognition and heterogeneous diagnostic pathways underscore the need for improving clinical awareness and implementing clearer diagnostic algorithms.

## Zusammenfassung

**Hintergrund:**

Die kutane Leishmaniose (KL) ist eine in Südeuropa wenig erkannte parasitäre Erkrankung, bei der heterogene klinische Präsentationen und eine begrenzte Vertrautheit der Kliniker zu einer verzögerten Diagnosestellung beitragen können.

**Methode:**

Wir führten eine 15-jährige retrospektive Fallserienstudie an einem Tertiärversorgungszentrum auf Kreta (Griechenland) durch, um die epidemiologischen, klinischen, laborchemischen und therapeutischen Charakteristika bestätigter KL-Fälle zu beschreiben.

**Ergebnisse:**

Es wurden fünfundzwanzig Patientinnen und Patienten identifiziert, darunter zwei mit gleichzeitiger viszeraler Leishmaniose. Die meisten stellten sich mit solitären, schmerzlosen Läsionen an exponierten Körperstellen vor. Die mediane Dauer der Hautläsionen vor Diagnosestellung betrug 327 d und verdeutlicht eine erhebliche diagnostische Verzögerung. Die PCR erwies sich als sensitivstes diagnostisches Verfahren, gefolgt von Histopathologie und Kultur; die Speziesidentifikation bestätigte *Leishmania tropica* in ausgewählten KL-Fällen.

Die Therapieansätze variierten und umfassten systemische Medikamente, topische Behandlungen und Beobachtung. Versagens- bzw. Rezidivraten lagen bei 12% bzw. 16% und traten überwiegend bei Patientinnen und Patienten mit relevanten Komorbiditäten oder Immunsuppression auf.

**Schlussfolgerung:**

Die Insel Kreta stellt weiterhin ein persistierendes Epizentrum der KL in Griechenland dar, mit einer niedrigen scheinbaren Inzidenz, jedoch bemerkenswerter klinischer Komplexität. Verzögerte Diagnosestellung und heterogene diagnostische Vorgehensweisen unterstreichen die Notwendigkeit einer verbesserten klinischen Aufmerksamkeit sowie klarerer diagnostischer Algorithmen.

## Introduction

Cutaneous leishmaniasis (CL) is an anthroponotic parasitic skin disease caused by *Leishmania (L.) *spp. and transmitted by phlebotomine sandflies. Although it is endemic in several Mediterranean countries, human CL remains relatively uncommon in Greece [1]. The island of Crete, however, has long been recognised as a receptive environment for leishmaniasis transmission, with both *L. tropica* and *L. infantum* identified in human infections [2]. Isolated reports of locally acquired and imported cases of CL linked to Crete suggest that transmission persists, albeit at low apparent incidence, due to the presence of sandfly vectors on the island [3], [4]. 

Despite ongoing autochthonous transmission, contemporary data describing the clinical and epidemiological profile of human CL in Crete remain limited. This study aimed to report the epidemiological and clinical characteristics, diagnostic tools, treatment, and outcome of CL among patients treated at a tertiary care hospital in Crete, Greece, over a 15-year surveillance period.

## Methods

This retrospective case-series study was conducted at the University Hospital of Heraklion, a 750-bed tertiary care center serving more than 55,000 inpatients annually as the referral hospital for the island of Crete. The study evaluated all patients with a well-established CL diagnosis who were treated at the study center between January 2004 and December 2018. The patients’ electronic and written charts were reviewed for demographic characteristics, underlying diseases, clinical manifestations, laboratory parameters, and treatment outcomes.

An index patient was diagnosed based on the presence of characteristic lesions — chronic papules, nodules, or ulcers with raised indurated margins — occurring in individuals with relevant epidemiologic exposures. Although clinical findings may be suggestive, definitive diagnosis requires demonstration of Leishmania parasites by direct microscopy of lesion material or confirmation by PCR, which provides higher sensitivity and enables species identification [5], [6]. Serological tests contribute little due to weak antibody responses, and current guidelines emphasize integrating compatible clinical features with epidemiological risk and parasitological or molecular evidence for confirmation [5].

Response to treatment was assessed clinically. Successful response (clinical cure) was defined as the complete re-epithelialization of all ulcers without residual crusts, resolution of induration and inflammation and no appearance of new lesions achieved by 3 months after completion of treatment. Failure to respond was defined as no reduction or an increase in lesion size by 4–6 weeks, or incomplete healing (persistent ulceration, induration, or inflammation) by 3 months after therapy, or development of new lesions after treatment. 

CL relapse was defined as the reappearance of active cutaneous lesions at the site of previously healed lesions within 24 months after documented clinical cure, characterized by complete re-epithelialization and resolution of inflammation. Relapse occurs after a symptom-free interval and in the absence of new exposure to the parasite, indicating renewed local parasitic activity rather than reinfection or initial treatment failure [7]. 

The study was approved by the hospital’s institutional review board (approval no. 12/14-09-2016).

## Results

**Figure 1:**
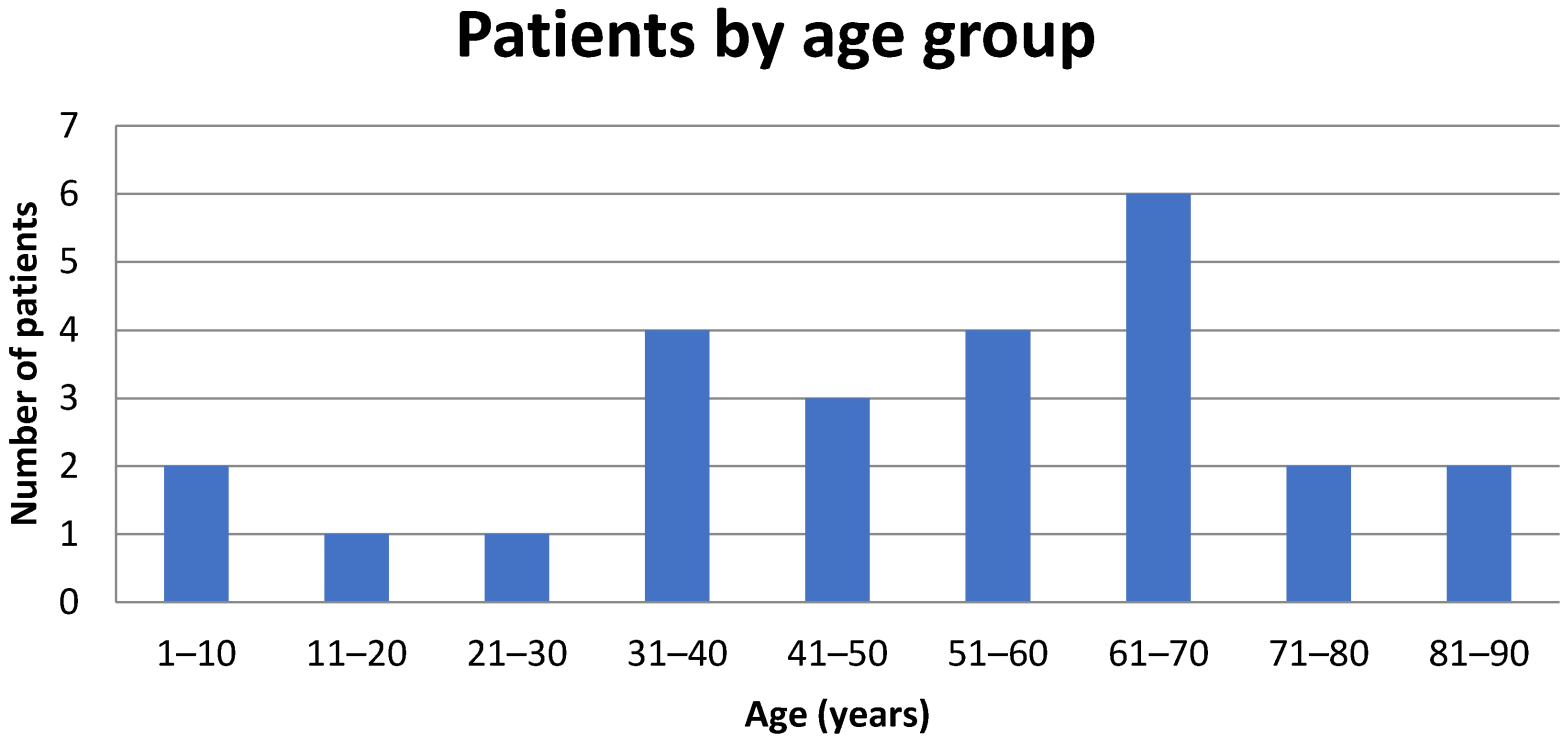
Distribution of patients with cutaneous leishmaniasis according to age

Over 15 years, 23 patients were identified with confirmed CL, while 2 additional patients presented with concomitant CL and visceral leishmaniasis (VL). Notably, in one of the patients with dual infection, the diagnosis of CL had been established one year earlier, but the patient had declined treatment at that time. The mean age of the cohort was 50.3 (SD 22.1) years; 14 (56%) patients were male (age distribution Figure 1 (Fig. 1)) . Of the 25 patients, 22 were of Greek origin, while one was from Albania, one from Spain, and one from Afghanistan. The Afghan patient was a 13-year-old male refugee who had recently arrived in Greece [4]. Fourteen patients (56%) resided in rural areas, whereas 11 (44%) lived in urban settings. Among those with available information on animal exposure, 8 of 19 patients (42%) reported frequent contact with animals, primarily stray or domestic dogs.

Regarding comorbidities, one patient of each had a history of splenectomy, multiple myeloma, Crohn’s disease and was receiving adalimumab, rheumatoid arthritis treated with methotrexate and hydroxychloroquine, stage-4 chronic kidney disease, chronic hepatitis B virus (HBV) infection without cirrhosis, and diabetes mellitus. Notably, the asplenic patient presented with concomitant VL and CL. Four patients reported CL in the past, and three patients reported a family history of the disease.

Twenty-four patients sought medical evaluation for skin lesions, whereas one patient presented with generalized weakness. Twenty-two patients were managed by the Dermatology Department, two by the Internal Medicine Department, and one by the Otorhinolaryngology Department; the latter had lesions of the nose. Twenty patients required hospitalisation, while five were evaluated and treated as outpatients. Among hospitalised patients, the mean length of hospital stay was 10.4 days.

The main clinical and laboratory findings are summarized in Table 1 (Tab. 1). The median duration of skin lesions at diagnosis was 327 days (range: 21–1400 days). Overall, 13 patients presented with a single lesion, while one patient had five lesions. Among the 14 patients with available data regarding pain, 12 patients reported painless lesions, whereas 2 described associated pain.

Diagnosis was mostly established by PCR specific for *Leishmania* spp., which was positive in 16 of 25 patients. Histopathological identification of the parasite or Leishman–Donovan bodies on skin biopsy was achieved in 13 of 25 patients. In two cases, *Leishmania* parasites were detected in bone marrow biopsy specimens, one of which was the patient with rheumatoid arthritis. Culture of the parasite was positive in 7 of 11 samples submitted. It is noted that species identification was performed in a subset of cases. *L. infantum* was identified in three cases, *L. tropica* in two. Serology was positive in 6 of 15 tested patients. At the time of diagnosis, one patient had a documented co-infection with *Mycobacterium tuberculosis*. 

Regarding treatment, three patients received liposomal amphotericin B (L-AMB; cumulative dose 21 mg/kg). Five patients were treated with meglumine antimoniate, either as monotherapy or in combination with other modalities. Three patients received fluconazole, and 12 patients underwent topical therapy, either alone or alongside systemic treatment. Notably, five patients received no treatment: two declined therapy, and three experienced spontaneous lesion regression. 

It is worth noting that one patient who initially started on meglumine antimoniate required a change in therapy due to acute pancreatitis, while another patient receiving fluconazole discontinued treatment owing to worsening renal function.

Treatment failure occurred in three cases. In one case, involving the patient with Crohn’s disease receiving adalimumab, rescue therapy with L-AMB was initiated. In another case, the patient with underlying chronic kidney disease required subsequent cryotherapy. In the third case, biopsy revealed the presence of basal cell carcinoma adjacent to the leishmanial lesion.

Relapse of CL occurred in four patients. These included the patient with Crohn’s disease previously treated with adalimumab, one patient with diabetes mellitus, and one with chronic HBV infection. One of these individuals experienced two relapses. All relapsed patients were successfully managed with systemic therapy—pentamidine, meglumine antimoniate, or L-AMB—with one case also receiving adjunctive cryotherapy. 

## Discussion

In this 15-year case series from Crete, we describe the epidemiological, clinical, laboratory, and therapeutic characteristics of 25 patients with CL, including 2 with concomitant VL. Although primarily clinical, these findings can also have implications for clinician awareness and early recognition pathways, which are relevant to infection prevention frameworks. Our findings complement earlier work from the island, which documented re-emergence of both VL and CL, identified *L. infantum* as the main agent of zoonotic VL alongside a high prevalence of infection in dogs, and demonstrated local CL due to *L. tropica* and abundant sandfly vectors [2]. Recent One Health-oriented overviews from Greece highlight that the country remains endemic for leishmaniasis, with human, animal, and entomological data all pointing to sustained transmission and the need for integrated surveillance [8], [9]. In this context, clinical datasets from tertiary-care hospitals can support earlier recognition and complement national surveillance systems by informing healthcare-based awareness strategies.

The age distribution and predominance of adults in our cohort are compatible with patterns described for CL in Old-World settings, where exposure to sandflies in rural and peri-domestic environments plays a central role [10], often affecting adults due to occupational and outdoor activities. The predominance of rural residence found by Christodoulou et al. proved that Crete is an endemic zone with increasing human VL cases and re-emergent CL [2]. Together with national surveillance data showing that both VL and CL have been consistently reported over the period 2004–2018 [1], [5], our series fits into a broader picture of stable or re-emerging leishmaniasis in Greece. These epidemiological patterns underscore the importance for clinicians in endemic regions to maintain familiarity with CL presentations when assessing persistent or atypical cutaneous lesions.

Clinically, most of our patients presented with solitary, predominantly painless lesions on exposed sites (face and limbs), with ulcerative, nodular, or plaque-like morphologies. This is in line with the classical description of Old-World CL, and in particular CL due to *L. infantum* and *L. tropica* in the Mediterranean basin [10], [11]. Del Giudice et al. [11], reviewing CL due to *L. infantum*, reported mainly localized lesions on exposed areas in patients from Mediterranean countries, and de Vries and Schallig [10] summarize a broad spectrum of papulonodular and ulcerative lesions in Old-World CL. The relatively high proportion of facial lesions in our cohort is clinically important because of cosmetic impact and the potential for functional impairment, as also underlined in these reviews [10], [11]. From an infection-prevention perspective, the visibility and chronicity of these lesions create an opportunity for earlier identification within primary and secondary healthcare services.

A striking feature of our series is the long median duration of lesions (almost 11 months) before diagnosis. Narrative reviews have repeatedly pointed out that CL is often under-recognized and misdiagnosed in non-tropical or low-incidence settings, that lesions may be indolent, and that diagnostic delays of several months are common [10]. This delay has practical implications, including prolonged patient discomfort and increased risk of scarring [10], [11]. It also highlights the need for clearer diagnostic pathways and educational efforts within healthcare institutions to reduce repeated consultations, delayed referrals, and multiple specimen submissions. In our patients, systemic symptoms and laboratory abnormalities were generally mild or absent (as expected); however, the two cases with concomitant VL and CL underscore that clinicians should consider visceral involvement in patients with suspicious skin lesions and systemic signs, such as cytopenias, hepatosplenomegaly, or prolonged fever. The coexistence of cutaneous and visceral disease has been well described in *L. infantum* infection, particularly in Mediterranean Europe [2], [12]. Timely recognition of such presentations is relevant not only for individual patient safety but also for the development of efficient clinical workflows in healthcare settings.

Our diagnostic strategy relied mainly on PCR for Leishmania spp. and histopathology of skin biopsies, with culture and serology used less often. This approach is consistent with current recommendations summarized in recent comprehensive reviews, which emphasize that molecular methods on appropriate tissue samples have the highest sensitivity and allow species-level identification, whereas histology and smear microscopy are less sensitive but still useful, with serology playing a limited role in isolated CL [10]. The performance we observed (PCR-positive in the majority of those tested, histology confirming about half) falls within the range reported in the literature and reflects the realities of routine clinical practice [10]. Given that viable parasites may be present in biopsy material, and in light of documented laboratory-acquired Leishmania infections [13], [14], adherence to standard biosafety precautions is advisable during specimen handling in diagnostic laboratories.

Therapeutic management in our cohort was heterogeneous and included systemic agents (meglumine antimoniate, liposomal amphotericin B, fluconazole), topical therapies, and, in some cases, no specific treatment when spontaneous regression occurred. This variability mirrors what is seen in international practice and is well documented in recent reviews of CL treatment, which describe a wide range of systemic and local options, often with limited head-to-head comparative data and substantial regional variation [10], [15]. The studies by Burza et al. [10] and Shmueli and Ben-Shimol [15] both emphasize that antimonial drugs, amphotericin formulations, and other systemic agents are associated with significant toxicity and logistical challenges, and that treatment choice must balance efficacy, safety, practicality, and cost. Our observation of pancreatitis with meglumine antimoniate is consistent with the recognised potential for adverse events during anti-leishmanial therapy [12], [15], This heterogeneity also underscores the potential value of developing more standardized institutional treatment approaches in endemic areas.

Spontaneous regression of lesions in three of our patients who did not receive specific therapy is compatible with the natural history of many Old-World CL cases, particularly when lesions are few and the host is immunocompetent [10], [12]. The publications by de Vries and Schallig [10] and Burza et al. [12] both emphasize that management decisions in CL should be individualized, taking into account factors such as lesion number, anatomical site, the potential for disfigurement, and other clinical considerations. Our findings are in line with existing evidence suggesting that local or conservative management is appropriate for small, uncomplicated CL lesions in immunocompetent individuals, whereas systemic therapy is more often indicated for patients with multiple lesions, when cosmetically sensitive sites are involved, or other complicating features [10], [12], [15] Clearer clinical pathways may help standardize these decisions within healthcare systems.

Treatment failure and relapse in our series were uncommon but clinically meaningful, clustering mainly in patients with important comorbidities (Crohn’s disease on adalimumab, diabetes, chronic hepatitis B, chronic kidney disease). A recent systematic review by Santos et al. [16] shows that treatment failure and clinical relapse in leishmaniasis are multifactorial phenomena, influenced by both parasite-related and host-related factors. Their analysis highlights host immunological status as an important determinant of outcome. Additionally, reports focusing on patients receiving tumour necrosis factor-alpha antagonists in the Mediterranean have demonstrated that TNF-a blockade is associated with atypical clinical presentations and more complicated courses of leishmaniasis and recommend systemic therapy plus interruption of the biologic treatment (i.e., medication derived from living organisms and reproduced in living cells) until resolution [17]. The Crohn’s disease patient on adalimumab, who experienced failure and relapse, fits this pattern and underlines the need for a high index of suspicion and closer follow-up in patients receiving biologics n endemic areas [12], [17]. Notably, regarding one patient, adalimumab was discontinued after the diagnosis of CL. These observations highlight the importance of structured follow-up of immunocompromised patients, a group of particular relevance to infection prevention policies.

The association of relapse with chronic comorbidities in our cohort is also consistent with broader evidence that immunocompromised or otherwise vulnerable populations (e.g., extremes of age, HIV co-infection, chronic systemic disease) can have more severe or protracted leishmaniasis and may respond less favorably to standard regimens [12], [18], [16]. Castro et al. [18], in their collaborative retrospective study of “special populations” with CL (children=10 years and adults=60 years), showed that treatment outcomes and safety profiles can differ substantially from those in younger, otherwise healthy adults. Although our series did not specifically focus on such age-defined groups, it reinforces the concept that risk stratification based on host factors is essential when planning therapy and follow-up [12], [15], [18], [16]. Recognizing such vulnerable groups is also relevant for tailoring IPC-related monitoring in clinical environments.

From an epidemiological and public health standpoint, our results complement national data showing that human leishmaniasis remains an important issue in Greece [19]. Tzani et al. [1] analyzed surveillance data from 2004 to 2018 and concluded that both VL and CL retain public health relevance and that a sustainable action plan is needed for their management and control. Fotakis et al. [8] expanded on this by proposing a transition to a One Health surveillance system that integrates human, veterinary, and entomological information to better capture the true burden and guide interventions. Symeonidou et al. [9] provided the veterinary perspective, documenting widespread canine leishmaniasis and emphasizing the risk of further spread of VL. Our retrospective clinical data from Crete are in agreement with these observations and underscore that CL, although less frequently reported than VL in national statistics, can pose significant diagnostic and therapeutic challenges at the individual patient level. Sharing such hospital-based observations with regional surveillance bodies could improve timely recognition and guide prevention strategies. 

### Limitations

The retrospective design and relatively small sample size of the study limit the statistical power to identify independent predictors of outcome. Diagnostic and therapeutic strategies were not standardized and evolved over the 15-year study period. This reflects real-world practice but complicates direct comparison of regimens. 

Species identification was not systematically performed, although prior work from Crete has clearly demonstrated that *L. tropica* causes human CL on the island [2], [19], [4]. 

Follow-up was variable, so late relapses or long-term cosmetic outcomes may have been under-reported [7]. 

Finally, as a tertiary-care hospital series, our cohort may over-represent more severe or complicated cases compared with the full spectrum of CL in the community. Nevertheless, such hospital-based experiences can inform clinical awareness, early diagnostic approaches, and safe specimen handling procedures within healthcare services.

Conclusion

Our findings add clinically meaningful information on leishmaniasis in Crete, highlight the chronic and often indolent nature of CL lesions, the importance of considering CL in the differential diagnosis of persistent facial or limb lesions in residents of or visitors to/from endemic areas, the complexity of treatment decisions in the absence of species-specific evidence, and the increased risk of treatment failure and relapse in patients with significant immunosuppression or comorbidities. These conclusions are aligned with current international reviews on CL diagnosis and management and with national calls for strengthened, One Health–based surveillance and control strategies [1], [8], [10], [9], [12], [15], [18], [16]. In addition, our data suggest practical areas where healthcare settings may enhance clinical pathways and diagnostic awareness relevant to infection prevention.

Future work in Greece and other Mediterranean countries should include prospective, multicenter studies with standardized diagnostic algorithms, routine species identification, and protocolized treatment and follow-up, building on collaborative approaches such as those used by Castro et al. [18]. Particular attention should be given to immunocompromised and otherwise vulnerable patients, who, as existing reviews and our series both suggest, may require tailored therapeutic strategies and closer surveillance [12], [15], [18], [16].

## Conclusions

Our findings add clinically meaningful information on leishmaniasis in Crete, highlight the chronic and often indolent nature of CL lesions, the importance of considering CL in the differential diagnosis of persistent facial or limb lesions in residents of or visitors to/from endemic areas, the complexity of treatment decisions in the absence of species-specific evidence, and the increased risk of treatment failure and relapse in patients with significant immunosuppression or comorbidities. These conclusions are aligned with current international reviews on CL diagnosis and management and with national calls for strengthened, One Health–based surveillance and control strategies [1], [8], [10], [9], [12], [15], [18], [16]. In addition, our data suggest practical areas where healthcare settings may enhance clinical pathways and diagnostic awareness relevant to infection prevention.

Future work in Greece and other Mediterranean countries should include prospective, multicenter studies with standardized diagnostic algorithms, routine species identification, and protocolized treatment and follow-up, building on collaborative approaches such as those used by Castro et al. [18]. Particular attention should be given to immunocompromised and otherwise vulnerable patients, who, as existing reviews and our series both suggest, may require tailored therapeutic strategies and closer surveillance [12], [15], [18], [16].

## Notes

### Competing interests

The authors declare that they have no competing interests.

### Authors’ ORCIDs:

- Aristodimou A: https://orcid.org/0009-0003-6567-1999
- Kritsotakis EI: https://orcid.org/0000-0002-9526-3852
- Antoniou M: https://orcid.org/0000-0002-4349-494X
- Krasagakis K: https://orcid.org/0000-0003-2197-2530
- Evangelou G: https://orcid.org/0000-0003-4468-446X
- Gikas A: https://orcid.org/0000-0002-8455-9631

### Ethical approval 

The study was approved by the hospital’s institutional review board (approval no. 12/14-09-2016).

### Funding

None

## Figures and Tables

**Table 1:**
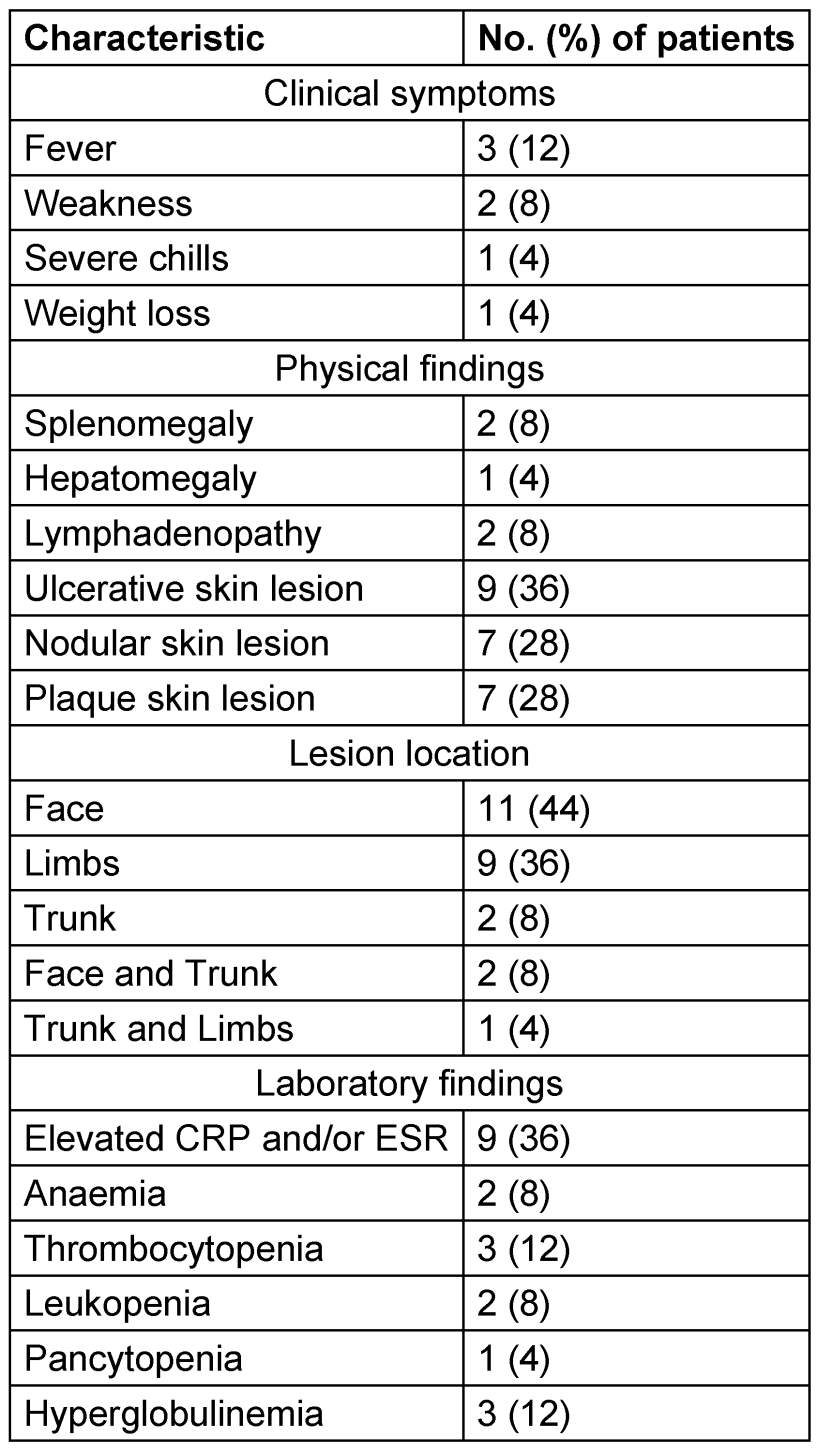
Clinical and laboratory characteristics of the 25 patients with cutaneous leishmaniasis
